# Diamond Model of Green Commitment and Low-Carbon Travel Motivation, Constraint, and Intention

**DOI:** 10.3390/ijerph19148454

**Published:** 2022-07-11

**Authors:** An-Jin Shie, You-Yu Dai, Ming-Xing Shen, Li Tian, Ming Yang, Wen-Wei Luo, Yenchun Jim Wu, Zhao-Hui Su

**Affiliations:** 1 College of Business Administration, Huaqiao University, Quanzhou 362021, China; ajshie@hqu.edu.cn (A.-J.S.); zhaohui@hqu.edu.cn (Z.-H.S.); 2 School of Economics and Management, Huaiyin Normal University, Huai’an 223309, China; 3 International College, Krirk University, Bangkok 10220, Thailand; starshenmingxing@126.com (M.-X.S.); mingyang116@163.com (M.Y.); 4 School of Big Data, Fuzhou University of International Studies and Trade, Fuzhou 350200, China; 5 International Business School, Shandong Jiaotong University, Weihai 264209, China; 223014@sdjtu.edu.cn; 6 School of Economics and Management, Harbin University of Science and Technology, Harbin 150080, China; 7 School of Marxism, Huaqiao University, Quanzhou 362021, China; wwluo@hqu.edu.cn; 8 Graduate Institute of Global Business and Strategy, National Taiwan Normal University, Taipei 106, Taiwan

**Keywords:** low-carbon travel, green commitment, low-carbon travel motivation, low-carbon travel constraint

## Abstract

Although consumers generally accept and care about environmental issues, consumers have not adjusted their behavior accordingly. Based on the diamond model theory, this study proposes and tests the direct impact of personal green commitments on low-carbon travel motivation and constraint, and the possibility of subsequent low-carbon travel intention. According to the results of 358 valid questionnaire surveys, this study shows that green commitments positively affect the low-carbon travel motivation and intention, while negatively affecting the low-carbon travel constraint. The low-carbon travel motivation has some mediating effects. The research results can be used as a reference by relevant managers of the tourism industry to make changes in the content of travel services that are more suitable for specific populations.

## 1. Introduction

As tourists’ travel behaviors generate large amounts of carbon dioxide emissions, visitors are aware of the environmental threats caused by carbon emissions [1], and motivate visitors to focus on reducing the specific carbon footprint of the holiday action [2,3]. Wen et al. [4] believed that tourists have generally accepted the concept of reducing greenhouse gases and self-carbon emission reductions for a sustainable environment. The focus of their practice is on energy conservation and carbon reduction. Low-carbon tourism not only meets tourists’ preference for energy-saving and carbon-reducing (ESCR) tourism, but also changes the high-carbon emissions of some tourists [3], and enables tourists to contribute to environmental protection through ESCR activities. Therefore, low-carbon tourism focuses on reducing carbon emissions, reducing carbon footprints during tourism, and encouraging visitors to use less energy to reduce environmental impacts during travel and accommodation [5].

On the whole, low-carbon tourism has the characteristics of low energy consumption and low pollution for the sightseeing environment and is a deep concept of environmental tourism [6]. Low-carbon tourism may not be suitable for every kind of sightseeing situation, but low-carbon tourism has considerable potential for development in most of the world’s domestic or short-term fake tourism. For example, Ceron and Dubois [7] pointed out that even if France reduces its carbon dioxide emissions by a quarter of the current tourism industry by 2050, tourism is clearly feasible. Hsiao [8] surveyed the poor and dependent tourism industry’s tourism market to transform into low-carbon tourism on the premise of environmental conservation, and the number of tourists has grown. Oh, Assaf and Baloglu [2] argued that slow travel is a viable strategy for low-carbon tourism in the future and that tourists engaged in slow travel will make the tourism industry more sustainable.

Low-carbon travel motivation comes from the internal driving force of tourists, or from the external attraction and attributes associated with low-carbon tourism, which explains why tourists engage in low-carbon tourism [9]. In contrast, the low-carbon travel constraint explains why tourists do not engage in low-carbon tourism in terms of their personal internal, interpersonal relationships, structure, and non-travel options. Safari, et al. [10] believed that commitment is influential in a person’s green travel behavior. Consumers’ green commitments to the environment is considered an important factor for predicting environmentally responsible behavior in traveling (Safari, Salehzadeh, Panahi and Abolghasemian [10]). Although some studies have been conducted in the field of tourists’ low-carbon travel behavior [11,12,13,14,15,16,17,18], additional empirical evidence is needed to be gathered before we can be sure of the causal impacts of the results. Especially, there is a need to focus on the mechanisms through which various personal and contextual antecedents influence tourists’ low-carbon travel behavior. The contribution of this study is to analyze the simultaneous impact of consumers’ green commitment on low-carbon travel intention with respect to the roles of low-carbon travel motivation and low-carbon travel constraint as the mediator variables.

Currently, environmentalism is upsurging increasingly on the political and social agenda. Although consumers generally accept and pay attention to environmental protection issues, consumers have not adjusted their behavior. From previous studies, the research questions and gaps are described in three aspects:

(a) Studies have seldom studied consumers’ green commitments, and low-carbon travel motivation and constraint;
(b) The lack of concentration on studies about the relationship between consumers’ green commitments on low-carbon travel motivation and constraint, and the possibility of subsequent low-carbon travel intention;
(c) The lack of a research model to incorporate consumers’ green commitments and low-carbon travel theories.

To address the research questions above, this study **aims** to explore whether exists the positive impact of consumers’ green commitments on low-carbon travel motivation and constraint, and improving the possibility of subsequent low-carbon travel intention by the proposed diamond model is initialized from an environmental protection idea; “lucid waters and lush mountains are invaluable assets”, said by Chinese President Xi Jinping. The diamond model consists of consumers’ green commitments, low-carbon travel motivation, travel constraint, and travel intention. The specific **objectives** of the present study are, therefore:

(a) Developing a theoretical research model and measurement scale by using consumers’ green commitments, and low-carbon travel theories;
(b) Exploring the relationship among consumers’ green commitments, low-carbon travel motivation, travel constraint, and travel intention by incorporating a consumers’ green commitments and low-carbon travel measurement scale;
(c) Testing consumers’ green commitments whether existing the positive moderating effect on the relationship between low-carbon travel constraint and low-carbon travel intention.

Finally, the remainder of this paper is organized as follows. Section 2 presents a comprehensive related literature on green commitment, low-carbon travel motivation, low-carbon travel constraint, and low-carbon travel intention. In Section 3, the proposed design methodology of the diamond model is described. In Section 4, the statistical analysis results of the proposed diamond model are presented. Then, the conclusions are summarized with the main findings, implications, and suggestions for future research in Section 5.

## 2. Literature Review

### 2.1. Low-Carbon Travel Motivation

Travel motivation is a series of needs that presuppose individuals to a particular tourism activity [19], primarily influenced by perceived costs and values [20]. Vujičić, et al. [21] defined tourist motivation as a fully integrated network of biology and culture, giving value and direction to tourism choices, behaviors, and experiences. Xu, et al. [22] argued that tourist motivation is the emotional and cognitive stimulus that causes behavioral intent. Dai, et al. [23] pointed out that understanding tourists’ Low-carbon travel motivation will help us identify the factors that drive tourists to participate in low-carbon travel.

Kuo and Dai (2015) and Zhang, Chen, Du and Wang [6] suggested that tourists participating in low-carbon tourism should not only reduce carbon dioxide or their carbon footprint, but also promote personal health, protect and sustain the tourism environment and recreational resources, and reflect the environmental problems brought by individuals and society. Horng, et al. [24] proposed the motivation for visitors to visit an ESCR activity: knowing new ESCR measures, obtaining more ESCR information, and expanding more interest ESCR issues, inspiring the willingness to save energy and reduce carbon, and exploring unknown ways to save energy and reduce carbon. Horng, Hu, Teng and Lin [24] and Li, Lo and Guo [17] believed that tourists’ environmental protection behavior is clearly driven by responsibility. Hu, et al. [25] and Li, et al. [26] studied people’s consumption behaviors on green diets, and suggested that moral identity satisfies individuals’ self-fulfillment.

In addition, Kim, Filimonau and Dickinson [3] argued that slow-moving travel has low-carbon potential, and they noted that environmental issues have led some interview participants to decide to reduce the number of flights or refrain flight altogether. Visitors’ environmental awareness is not only communicated to relatives and friends, but also positively and immediately leads to a reduction in the tourism carbon footprint. Visitors’ awareness of environmental shocks is increasing [5], and Jarratt and Davies [1] noted that tourists who are not often engaged in tourism activities are more willing to reduce the frequency of travel. Most tourists are willing to reduce carbon emissions during holidays. A lower carbon footprint is an added benefit to slow travel visitors, but it is not the main motivation for everyone [2].

### 2.2. Low-Carbon Travel Constraint

Although tourism has become a popular social phenomenon, there are still many reasons why people cannot travel extensively or not at all [27]. These reasons are specifically called travel constraints. Travel constraints are multi-faceted concepts that refer to factors that prevent or reduce the frequency, proportion, or fun of individuals engaging in specific activities [16,18,28,29]. Ying, Tang, Wen, Ye, Zhou and Li [28] argued that travel constraints are a key factor in preventing people from starting or continuing to travel. Wen, Huang and Goh [4] defined travel constraints as factors that inhibit travel continuity, lead to the inability to travel, result in the inability to maintain or increase travel frequency, and have a negative impact on tourism quality.

Dällenbach [30] believed that tourists’ environmental awareness is not easy to turn into environmentally friendly behavior when encountering travel holidays and climate change. Kuo and Dai [31] initially proposed that tourists’ low-carbon travel constraints include: no sustainable environmental concept, insufficient low-carbon tourism information, unreasonable travel prices, inconvenient travel, inconvenient travel and transportation, insufficient time and funds, unhealthy body, and tourism industry are unwilling to cooperate. Jarratt and Davies [1] pointed out that most normal international tourists are aware of global environmental issues caused by carbon dioxide emissions, but are reluctant to change their tourism behavior. Horng, et al. [32] mentioned that energy conservation and carbon reduction may make tourism activities uncomfortable and inconvenient. Horng, Hu, Teng and Lin [24] mentioned that tourists generally do not believe that tourism and related measures cause serious harm to the environment. In short, identifying low-carbon tourism barriers to action is a key step in promoting behavioral change.

Slow travelers realize that tourism activities have an impact on climate change, but engaging in low-carbon activities (such as reducing flight travel) is difficult and uncomfortable. Dällenbach [30] pointed out that the three obstacles affecting tourists’ changing behavior are: (i) no alternative transportation is considered; (ii) holidays are important; and (iii) responsibility lies with others. Hsiao [8] mentioned that people lack confidence in whether their actions change, and most people find it more difficult to escape from a high-carbon lifestyle. Kim, Filimonau and Dickinson [3] pointed out that tourists will continue to engage in air travel for various reasons, emphasizing that personal interests will not be easily abandoned. Some visitors are conservative about the environmental impact of air travel because they are driven by family and friends. Some participants use “no choice” as a general tourism decision, which benefits individuals but violates public interest. Oh, Assaf and Baloglu [2] mentioned that some participants have doubts about the scientific nature of climate change, lack of awareness of climate change, and even think that they can act responsibly at home or in other areas, and there is no need to change the vacation arrangements. The above tourist opinion may be because tourists believe that tourism is an irregular activity. Reducing greenhouse gas emissions should be the responsibility of the public rather than a personal responsibility, making them more inclined to spend energy on travel [33].

### 2.3. Green Commitment

The concept of commitment is primarily used to explain a consistent pattern of behavior among individuals and to express a willingness to engage mentally and physically to participate in an activity [34]. Su, et al. [35] defined commitments in terms of attitudes and behaviors. According to Buchanan [36], although the promise does not involve how and why individuals begin to participate leisurely, once it is inspired it will indeed indicate the cause of behavioral consistency. Andrade, et al. [37] therefore believed that the application promise is more effective in explaining the obstacles to the negotiation process.

Longoni, et al. [38] argued that the basis of individual green commitment is its attitude, perceived effectiveness, past green behavior, and future intentions to engage in green behavior. Consumers’ green commitments describe their attitudes toward green consumption choices [39]. A verbal commitment is when an individual is prepared to perform something or show an intention for environmental benefit [40]. In short, the green commitment can be seen as the consistent behavior of individuals facing green environmental issues.

This study uses green commitment as an early cause of low-carbon travel motivation and low-carbon travel constraint. Endrejat, et al. [41] argued that consumers have more commitment to environmental behavior and they have a stronger environmental motivation. Al-Swidi and Saleh [42] clearly pointed out that environmentalists are more motivated to purchase environmentally certified products. In contrast, since green commitment represents a consistent behavior of individuals on environmental issues, when visitors have a high degree of green commitment, their participation in environmental protection will be less restricted by perceptual hindrance. In other words, visitors’ green commitments effectively reduces perceptions of low-carbon travel constraint. Based on the above literature, this study proposes the following hypotheses:

**H1.**  *Green commitment enhances low-carbon travel motivation.*

**H2.**  *Green commitment weakens low-carbon travel constraint.*

### 2.4. Low-Carbon Travel Intention

Behavioral intention is the most important factor between an individual’s inner thoughts and actual actions and refers to the willingness of a person to perform a given behavior [15,43]. Hu, Wu and Chen [15] and Kuo and Dai [31] pointed out that the most important factor in determining an individual’s behavior is his or her intention to engage in the act. This view was confirmed by Shin and Kang [44] to protect environmental behavior. Behavioral intentions have also received attention in research related to low-carbon tourism. Jarratt and Davies [1] and Li, Mao, Liu, Wei, Li and Yuan [26] pointed out that most tourists are willing to contribute to carbon reduction during holidays, for example, tourists are willing to spend more time on low-carbon activities, voluntarily purchase carbon taxes, choose low-carbon footprints, and travel locally. Kuo and Dai [31] pointed out that tourists are willing to engage in low-carbon tourism in the future and recommend relatives and friends to engage in low-carbon tourism. Horng, Hu, Teng and Lin [24] investigated the willingness of tourists to engage in various ESCR activities in tourist destinations, accommodations, and restaurants.

Since consumers’ green commitment is to show their readiness to be green or to show green intent for environmental benefits [15,18,39], this study used low-carbon travel intention as a result of a green commitment. According to the self-completion theory (SCT), individuals who commit to a given goal (such as recognizing a green lifestyle) are able to engage in various activities to achieve their goals [38]. Lalot, Quiamzade, Falomir-Pichastor and Gollwitzer [39] believed that the more a person commits to environmental issues, the greener consumer behavior will be. For example, some consumers show a high degree of green commitment to organic and origin-label foods [43]. Based on the above literature review, this study proposes the following hypothesis:

**H3.**  *Green commitment enhances low-carbon travel intention*.

This study uses low-carbon travel intention as a result of low-carbon travel motivation and low-carbon travel constraint. Tan and Lin [45] pointed out that travel motivation has been widely used to explain leisure behavior and intentions. The influence of travel motivation on travel intentions is demonstrated by cruise tourism and the willingness of visitors to revisit [4,15]. Conversely, travel constraint is a key factor in preventing people from travelling [46,47]. People often use their personal, interpersonal, and structural factors to reduce their willingness to travel. Travel constraints have been shown to have a negative impact on travel intention [4]. Based on the above literature review, this study proposes the following hypotheses:

**H4.**  *Low-carbon travel motivation enhances low-carbon travel intention*.

**H5.**  *Low-carbon travel constraint weakens low-carbon travel intention*.

### 2.5. Moderating Effect of Green Commitment

Commitment is expected to put individuals in a state of defensive motivation, and with a high degree of commitment, this defensive motivation will facilitate selective cognitive processing of messages to filter out issues that threaten individual attitudes [48]. High commitments have led to environmental actions that require significant resources (finance, manpower, and skills), while low commitments indicate that people have not continued to act [49]. Just as environmentalists use energy conservation and waste recycling as a self-symbol [38], a low-carbon tourist will act as a self-symbol to reduce the impact and positive behavior of the tourism environment.

Consumer research has tested the promised regulatory effects. Zhao, et al. [50] pointed out that the main effect of the promise is to make behavior and cognition more resistant to change. Su, Yang and Huang [35] believed that the more committed people will recognize and resist change. Lalot, Quiamzade, Falomir-Pichastor and Gollwitzer [39] confirmed that most consumers show partial commitment to green issues, followed by consumers with more or very high green commitments, and a few consumers have low or no green commitment. Do Khare [51] pointed out that although people without environmental commitments claim that they know environmental issues and are concerned about environmental issues (perceived effectiveness, actions, environmentally friendly purchases, recycling, resource conservation, willingness to pay, and more efforts to protect the environment) they maintain a very negative stance. In contrast, green actors have a favorable position in all environmental issues.

In light of commitment as consistent in behavior, this study should focus on how green commitments affect the inconsistencies between low-carbon travel constraint and low-carbon travel intention. We reason that, based on environmental interests, tourists’ green lifestyles on weekdays can overcome the obstacles they face in low-carbon tourism. Tourism is a part of life; visitors are more or less environmentally motivated, have environmental behavior in the past, and have environmental behavior intentions in the future. These commitments to the environment are also practiced in low-carbon tourism. In short, visitors’ green commitments are able to moderate the relationship between low-carbon travel constraint and low-carbon travel intention, and even have a reversal effect. Therefore, this study proposes the following hypothesis:

**H6.**  *Green commitment will moderate the relationship between low-carbon travel constraint and low-carbon travel intention*.

### 2.6. Research Structure

**Figure 1:**
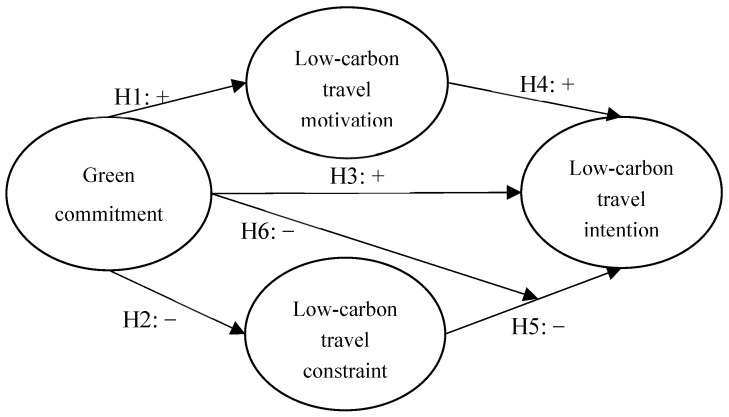
Diamond of this study.

This study explores the causal relationship between low-carbon travel motivation, low-carbon travel constraint, green commitment, and low-carbon travel intention. Green commitments refer to the attitudes of tourists towards low-carbon travel activities. Low-carbon travel motivation refers to a series of driving forces for tourists to favor low-carbon tourism activities. Low-carbon travel constraint is a factor that prevents or reduces tourists’ participation in low-carbon travel activities. Low-carbon travel intentions are the willingness of tourists to engage in low-carbon travel activities in the future and recommend others to work together. In particular, the existence of a green commitment moderates the relationship between low-carbon travel constraint and low-carbon travel intention. The concept of Porter’s diamond model theory is used to present the relationships between the four constructs, as well [52]. The relationships proposed in this study are shown in Figure 1.

## 3. Research Method

This section provides a concise and precise description of the experimental results, their interpretation, as well as the experimental conclusions that can be drawn.

**Green commitment** measurements were adopted from Longoni, Gollwitzer and Oettingen [38], with a total of 19 questions. **Low-carbon travel motivation** measurements were taken from [14] and Dai [9] and Dai, Shie, Chu and Wu [23] with 24 questions. **Low-carbon travel constraint** measurements were adopted from Dai [9] and Dai, Shie, Chu and Wu [23], with 23 questions. **Low-carbon travel intention** was adopted from Kuo and Dai [31] and Kuo and Dai [11], with a total of four questions. These four test items have been studied in the past to show their statistical reliability. The researchers designed the questionnaire as a 5-point Likert scale from 1 (strongly disagree) to 5 (strongly agree) presented in Appendix A.

The researchers intend to further pre-test a sample of tourists in Weihai and Quanzhou, China. Weihai is known as the most beautiful coastal tourist city, one of the top 10 livable cities in China in 2022, and is praised as “the most suitable place for human habitation” by the United Nations [53]. Low-carbon tourists can be clearly obtained as cycling travelers in Weihai City. Quanzhou was listed on the world cultural heritage list in 2021. Quanzhou also is the most suitable city for elderly care [54]. As a world heritage site and an eco-livable city with slow movement, Quanzhou has strengthened the construction of a low-carbon society.

The pre-test was conducted by purposed sampling during 1–7 October 2019. In this study, a total of 200 questionnaires were distributed in the two places by using the method of field survey [23]. In order to ensure the validity of the questionnaire, this study provided small gifts to the respondents and finally recovered 200 valid questionnaires. Then, the investigators used exploratory factor analysis (EFA) to determine the dimensions of the measurement scales. This study used Cronbach’s alpha values to indicate the internal consistency of each factor [55]. The Cronbach’s α value for all factors needs to be greater than 0.6 to represent statistical internal consistency reliability [56,57,58]. Cronbach’s alpha value of green commitment is 0.838, low-carbon travel motivation is 0.871, low-carbon travel constraint is 0.867, and low-carbon travel intention is 0.745. This study excluded items with cross-loading, and the factor load of all retained measurement items needs to be greater than 0.5. The KMO of green commitment is 0.771, low-carbon travel motivation is 0.845, low-carbon travel constraint is 0.824, and low-carbon travel intention is 0.739.

The COVID-19 pandemic started in Jan 2020; to collect formal questionnaires, Scherr and Reinemann [59] suggested online panel surveys, which are characterized by targeted sampling and are particularly effective in achieving low-level relationships. The survey group must include individuals who have participated in low-carbon tourism for a period of time. Today, group surveys are widely used in tourism research, such as recreation activities [60] and cruise tourism [4]. Therefore, this study used random online group surveys to collect quantitative data from Weihai and Quanzhou.

Researchers viewed former low-carbon tourism activities or actors as low-carbon tourists, while those who have not previously had low-carbon tourism experiences w non-low-carbon tourists. Researchers used random online groups to recruit participants. The final sample included low-carbon tourists and non-low-carbon tourists. The subjects were asked whether they had previously engaged in low-carbon tourism. The sample of this study is intended to be compared with past studies, to understand the low-carbon tourism market, and to make the sample of this study reasonably present the profile of the target group.

## 4. Results

The researchers used the snowball sampling method [61] to ask some 5-star hotels in Weihai and Quanzhou to agree to set our questionnaire QR code at the front desk. There are three hotels in Weihai and five hotels in Quanzhou that participated in this study. The questionnaire was issued from 1–31 July 2020 by Wenjuanxing, one of the largest online survey sites in China. To improve the recovery rate, there were incentives after the completion of the questionnaire. To ensure the respondents’ reliability and validity, this study had an open-ended examination question, that is “Please list the three most famous tourist attractions in Weihai (Quanzhou)”. For visitors with local travel experience (more than 2 days) to Weihai or Quanzhou in the recent half-year, 384 copies were recovered, 26 were invalidated, and 358 remained valid questionnaires (51.1%). In the valid questionnaires, 150 had travel experience in Weihai and 208 had travel experience in Quanzhou.

Most of the population was female (214, 59.8%). The most common age was 21–30 years old (235, 65.6%), followed by 31–40 (63, 17.6%), 41–50 (48, 13.4%), under 20 (6, 1.7%) and more than 50 years old (6, 1.7%). There are 227 (63.4%) unmarried people, followed by married but no child (106, 29.6%), married with children (23, 6.4%), and others (2, 0.6%). The highest education level was college, university, and above (241, 67.3%), followed by high school (93, 26.0%), and middle high school (24, 6.7%). Most respondents had travel experiences in the past 1 year 1 to 3 times (116, 32.4%), followed by 0 (85, 23.7%), 4~6 times (68, 19%), more than 6 times (52, 14.5%). Most monthly incomes were RMB 4501~9000 (276, 77.1%), followed by 9001~13,500 (37, 10.3%), less than 4500 (37, 10.3%), and more than 13,500 (7, 2.0%). Since this study and the research by Dai et al. both focus on low-carbon travel, the sample representativeness of this study is consistent with that of Dai, Shie, Chu and Wu [23].

### 4.1. Confirmatory Factor Analysis

This study used a confirmatory factor analysis to deal with the co-variation relationship between measured variables and their latent variables, and examined the convergent valid and discriminate validity of the measurement model. Convergent validity means that the observational variables in the same construct are highly correlated with each other, so these observational variables can be used to measure the same construct [56]. In terms of the recommended value of the evaluation measurement model, Guan, et al. [62] suggested that items with too high residual values or too low factor loading after standardization should be deleted, and the factor loading after standardization should be above 0.45, the squared multiple correlations (SMCs) of each item should be at least 0.50 or more.

In view of this, according to the evaluation criteria of the indicators suggested by the above researchers, this study tested the load of each item factor in the measurement mode and the significance t value. When the load of the factor meets 0.50 or more, the SMC value meets 0.20 to 0.50, and each estimated parameter t value is greater than 1.96, it means that the measurement item has reached a significant level. Table 1 shows that the 70 observed variables all reach a significant level (t > 1.96, *p* < 0.05), and the estimated parameter factor loadings are higher than the 0.5 criteria, from 0.50 to 0.71. In addition, the average variance extracted (AVE) of each construct of this study is: 0.62 for low-carbon travel motivation, 0.59 for low-carbon travel constraint, 0.58 for green commitment, and 0.65 for low-carbon travel intention, which is both greater than the recommended value of 0.5 from Matthes and Ball [56]. The composite reliability (CR) for low-carbon travel motivation is 0.80, for low-carbon travel the constraint is 0.77, for green commitment it is 0.75, and for low-carbon travel intention is 0.64, which is greater than the recommended value of 0.6 from Matthes and Ball [56]. The AVE and CR results indicate that the internal consistency of the model is generally acceptable. Therefore, the measurement model of this study has convergent validity.

Discriminate validity refers to the measurement of two different constructs. If the correlation between these two constructs is very low after a correlation analysis, it means that the two constructs have discriminate validity [63]. In terms of the discriminate validity test, this study first considers that the root means square of AVE of each facet is greater than the number of correlation coefficients of each facet, and it must account for at least 75% of the overall comparison number [56]. After the correlation analysis in this study, there is a significant correlation between the various constructs of the measurement model: the discriminate validity test followed, and the analysis results show that each construct meets the criterion, which proves that the various constructs are correlated but not the same factors have discriminate validity (Table 2).

### 4.2. Hypothesis Test of Model Fit and Theoretical Structure Model

Guan, Gong, Xie and Huan [62] pointed out that the standard error of the basic fitness standard cannot be negative, the standard error must reach a significant level, and the absolute value of the correlation between the estimated parameters should not be too close, and the factor loading should preferably be between 0.50 and 0.95 and should not be a very high standard error. In addition, whether the single parameter of the model is suitable or not requires attention to the feasibility of the parameter estimate, the appropriateness of the standard error, and the statistical significance of the parameter reference value. The index value of the analysis is mainly based on: the two index values of NNFI and CFI are between 0 and 1, greater than 0.9 is a good fit range; the RMSEA value is 0.00–1.00, less than 0.05 is the best, 0.05–0.08 is a good fit; the chi-square freedom ratio is less than 2, less than 5 is a good range [64].

The hypothetical model in this study has a good fit (df = 2340, χ^2^ = 5616.58, χ^2^/df = 2.40 < 5, RMSEA = 0.070, NNFI = 0.81, CFI = 0.82), by the hypothetical structure model. The estimated values of the path parameters to verify the hypothesis of this study is as follows: when the t value of the parameter is greater than 1.65 (*p* < 0.05, one-tailed), the path is assumed to be established (Table 3). There are five hypothetical paths for the structural model, and finally four paths are established as follows: green commitment significantly positively affects low-carbon tourism motivation, and low-carbon tourism intention, and significantly negatively affects low-carbon tourism barriers; low-carbon tourism motivation significantly positively affects low-carbon tourism intentions. The above indicates that the green commitment does have a direct impact on the people’s motivation and intention to engage in low-carbon tourism, and the green commitment will indeed inhibit the public’s perception of obstacles to low-carbon tourism. The stronger the people’s motivation to engage in low-carbon tourism, the stronger their willingness will be. The above results indicate that the people’s willingness to engage in low-carbon tourism does require a variety of interlocking and indispensable factors.

### 4.3. Moderating Effects of Green Commitment (H6)

This study a used K-means cluster analysis to group means in the comparison analysis [12]. Using tourists’ green commitment scores, tourists were categorized into either a low green commitment group (*n* = 141) or a high green commitment group (*n* = 148). This study then further adopted PROCESS 3.4 in SPSS to test the path coefficients of the two models. In the low green commitment group, the path coefficients are 0.29 for green commitment to low-carbon travel motivation, −0.22 for green commitment to low-carbon travel constraint, 0.25 for green commitment to low-carbon travel intention, 0.45 for low-carbon travel motivation to low-carbon travel intention, and −0.06 for low-carbon travel constraint to low-carbon travel intention. The path coefficients are significant at the level of *p* < 0.05 expect the path coefficient of low-carbon travel constraint to low-carbon travel intention.

In the high green commitment group, the path coefficients are 0.47 for green commitment to low-carbon travel motivation, −0.10 for green commitment to low-carbon travel constraint, 0.22 for green commitment to low-carbon travel intention, 0.25 for low-carbon travel motivation to low-carbon travel intention, and −0.07 for low-carbon travel constraint to low-carbon travel intention. The path coefficients are significant at the level of *p* < 0.05 expect the path coefficients of green commitment to low-carbon travel constraint and low-carbon travel constraint to low-carbon travel intention.

**Figure 2:**
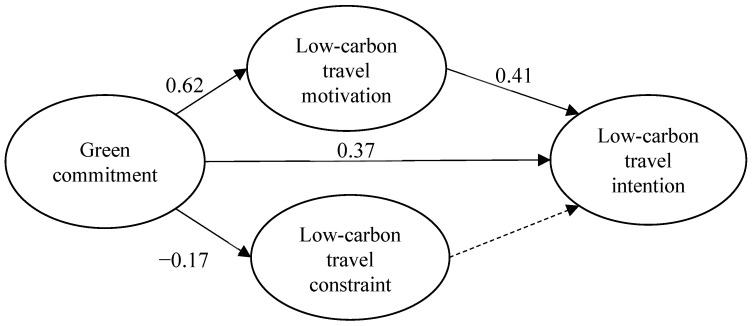
Final structural pattern diagram (*n* = 358). Note: the solid lines represent significant effects (*p* < 0.05), and the dashed lines represent no significant effects.

For the low-carbon travel tourists, their green commitment only had significant and direct positive effects on low-carbon travel motivation and intention but had no moderating effect on the relationship between low-carbon travel constraint to low-carbon travel intention. Therefore, H6 was not supported. Figure 2 illustrates the causal relationship between the various dimensions.

## 5. Discussion and Conclusions

### 5.1. Discussion

According to the result of the confirmatory factor analysis and hypothesis test of model fit and theoretical structure model above, the reliability and validity of scales in this questionnaire are in line with previous studies [9,14,23,37]. This represents that the scales are stable for the survey. Meanwhile, the hypothesis test results in the proposed diamond model are presented in Figure 1. First, the results of the **H1** and **H3** analysis revealed that consumer green commitment significantly positively affects low-carbon travel motivation and intention. The results of the **H2** analysis indicated that green commitment significantly minimizes low-carbon travel constraint. The results of **H4** analysis presented that low-carbon travel motivation significantly positively impacts on low-carbon travel intention. These two paths echo to Dai [9] in that low-carbon travel motivation and constraint can further forecast low-carbon travel intention and behavior. Since the researchers are the first ones to discuss the impact of green commitment on low-carbon travel motivation, constraint, and intention, green commitment plays has influence as seen in the study by Longoni, Gollwitzer and Oettingen [38].

Second, the results of the **H5** analysis presented that low-carbon travel constraint has no significantly negative influences on low-carbon travel intention, and the **H6** analysis indicated that consumer green commitment has no moderating effects on low-carbon travel constraint and intention.

In the lives of ordinary people, low-carbon tourism is not a well-known type of tourism. Driven by people’s personal awareness of green lifestyles, the motivation to engage in low-carbon tourism will increase. Green commitments significantly positively affect low-carbon travel motivation. At the same time, people with more significant green commitments engage in low-carbon tourism regardless of constraints. The green living style promised by people increase their willingness to participate in low-carbon tourism, which is the same as the results of Lalot, Quiamzade, Falomir-Pichastor and Gollwitzer [39] and Longoni, Gollwitzer and Oettingen [38]. Green commitments can also increase low-carbon travel intention through low-carbon travel motivation, that is, if people are more motivated to convert verbal commitments into specific behaviors before engaging in low-carbon travel activities, people will be more willing to engage in low-carbon tourism.

Additionally, a comparison of the strengths of the consumers’ green commitment used between this study and the previous studies is described clearly in Table 4 to better understand the research novelty. Previous studies focused on the rising of people’s environment awareness through environmental education to stimulate their protective actions. The previous studies lacked consideration of the consumers’ green commitments and low-carbon travel motivation and constraint. Specifically speaking, the research contributions of this study are: (1) the diamond model based on the perspective of consumers’ green commitments on low-carbon travel motivation and constraint were enriched; (2) a measurement scale for consumers’ green commitments for improving low-carbon travel attitude were enriched; (3) consumer green commitment increasing low-carbon travel motivation and intention and reducing low-carbon travel constraint were verified.

### 5.2. Conclusions

The comparison of travel motivation and travel constraint has been discussed and empirical in the research of many types of tourism in the past. However, in the past, most studies only started with motivation and constraints as research questions and did not discuss more antecedent influencing factors (e.g., consumers’ green commitment and low-carbon travel intention). Meanwhile, it was rare to study whether consumers’ green commitment improves low-carbon travel motivation or reduces constraints. Dai, Shie, Chu and Wu [23] suggested researchers adopting low-carbon travel motivation scale and low-carbon constraint scale to examine the relationships between tourists’ low-carbon travel motivation and low-carbon travel constraint and other variables.

The **theoretical contribution** of this study is to introduce the influence of green commitment on low-carbon travel motivation and constraint into the context of low-carbon tourism, and confirms it can improve low-carbon travel intention. At the same time, this study explored the antecedents and effects of green commitment, thereby confirming that low-carbon travel motivation can play a mediating role. These research results, whether for future travel motivations and travel constraints or low-carbon tourism context, provide the possibility of innovative theoretical construction. To summarize, this study enriches the research gaps from the previous studies on the low-carbon tourism issues.

Shih and Yao [76] and Li, Mao, Liu, Wei, Li and Yuan [26] believed that current social trends are ready to create a new market for low-carbon tourism products. This is particularly evident in the high percentage of global tourists engaged in tourism activities in Europe. Low-carbon tourism also has potential for development in the diversified short- to medium-range tourism in Asia. Participants in this study are aware of the benefits of energy-saving and carbon reduction. Although some people only have a slight understanding, it is obvious that people currently lack a bridge to act, and there are some influential discussions in society that deny the responsibility of tourism stakeholders. To a considerable extent, tourists are restricted by existing tourism products. For example, it is difficult to carry bicycles by train, but it is easy to rent bicycles on low-cost airlines, so low-carbon cycling tourists are more likely to engage in high-carbon airplane tourism [2].

It is generally believed that there are North–South differences in carbon emission treatment in China. The general situation is that the northern provinces of China attach great importance to the industrial development of the economy, but there are many population outflows. The southern provinces attach importance to market services and make progress in ecological concepts. However, the results of this study narrowed the gap between the north and the south.

The 2022 government work report of Weihai municipal government proposed to “adhere to the path of green and low carbon, and further promote the construction of ecological civilization” [53]. As a world heritage site and one of the earliest cities in China to implement “home-based elderly care”, Quanzhou has built 20 “low-carbon” pilot demonstrations in 2021. The policy is formulated from top to bottom. However, from the perspective of social practice, people’s spontaneous behavior from bottom to top is important. This study finds the significant path is “green commitment -> low-carbon tourism motivation -> low-carbon travel intention”. Dai, Shie, Chu and Wu [23] suggested that competent authorities of tourism destinations should actively educate tourists about environmental protection. Therefore, local governments can also consider enhancing peoples’ green commitment, low-carbon tourism motivation, and low-carbon travel intention through educational management measurements.

### 5.3. Limitations and Future Research Suggestions

This study mainly proposes a causal model for tourists from famous tourist attractions in Weihai, Shandong, and Quanzhou, Fujian, China. Due to time and financial considerations, only this city’s attractions were selected. It is recommended that follow-up studies expand the measurement range or target other tourists with more tourists. The low-carbon travel motivation and constraint questionnaire can extend to construct conceptualization, scale development, and validation, and further to cover a low-carbon travel market segmentation survey for urban tourist attractions. It is recommended that follow-up researchers increase interviews with the public, which may bring the results closer to practice. According to many literature reviews, people with more green commitments did not reduce their intention to engage in low-carbon tourism because of the obstacles to low-carbon tourism. It is recommended that follow-up research be considered to make the structure more perfect.

## Figures and Tables

**Table 1:** Confirmatory factor analysis of measurement scale (*n* = 358).

Constructs and Items	Standard Factor Loading	Standard Error	t-Value	Squared Multiple Correlations	Average Variance Extracted	Composite Reliability
**Low-carbon travel motivation (low-carbon travel in the “coastal livable city Weihai/world heritage site Quanzhou” would…)**					**0.62**	**0.80**
1. Help my health.	0.63	0.04	5.90	0.52		
2. Reduce the impact on tourism environment.	0.55	0.05	9.41	0.52		
3. Help me identify the concept of energy-saving and carbon-reducing (ESCR).	0.55	0.05	9.49	0.60		
4. Help me obtain low-carbon tourism knowledge.	0.54	0.05	7.44	0.72		
5. Reduce resource waste.	0.63	0.05	5.89	0.54		
6. Make me interested in the topic of energy saving and carbon reduction.	0.52	0.03	8.86	0.66		
7. Help me meet people with the same habit in ESCR.	0.54	0.03	7.45	0.58		
8. Help me respect nature.	0.52	0.03	6.88	0.57		
9. Allow me to be responsible for the environment.	0.55	0.05	9.60	0.51		
10. Provide opportunities for family/parental environmental education.	0.63	0.05	11.28	0.69		
11. Make me stay away from daily life/work environment.	0.57	0.03	9.88	0.64		
12. Make me avoid daily life/work stress.	0.58	0.03	8.11	0.69		
13. Make me obtain peace of mind and body.	0.56	0.05	9.62	0.76		
14. Make me contact my relatives and friends.	0.57	0.05	9.95	0.66		
15. Make me explore the humanities of travel destinations at a fixed place deeply.	0.56	0.05	7.67	0.63		
16. Make me experience convenient mass transportation (train, bus, and high-speed rail).	0.59	0.05	6.34	0.62		
17. Make me experience perfect bicycle or trail facilities.	0.60	0.05	14.34	0.58		
18. Help the industries have environmental labels (such as environmentally friendly hotels, environmentally friendly restaurants, and environmentally friendly vehicles).	0.54	0.05	7.51	0.60		
19. Help me to think environmental slogans and appeals of travel industries.	0.54	0.05	7.50	0.69		
20. Improve travel industries to provide ESCR measures.	0.53	0.05	9.02	0.64		
21. Help me buy environmentally friendly products.	0.63	0.05	5.94	0.62		
22. Help me purchase local products.	0.54	0.05	9.31	0.60		
23. Help me eat local foods.	0.59	0.05	8.24	0.66		
24. Help me experience the difference between low-carbon tourism and general tourism.	0.50	0.05	6.68	0.67		
**Low-carbon travel constraint (Not to low-carbon travel in the “coastal livable city Weihai/world heritage site Quanzhou” because…)**					**0.59**	**0.77**
1. My low-carbon tourism knowledge is not enough.	0.52	0.04	6.91	0.54		
2. I do not know how to engage in low-carbon tourism.	0.57	0.04	7.90	0.57		
3. My physical condition is not suitable for low-carbon tourism.	0.50	0.04	8.52	0.60		
4. I have no time for low-carbon tourism.	0.50	0.04	6.64	0.54		
5. I have no money for low-carbon tourism.	0.58	0.04	6.30	0.59		
6. I have no energy for low-carbon tourism.	0.51	0.04	6.74	0.65		
7. I have a lot of work or scholarship responsibility.	0.53	0.03	5.37	0.59		
8. I am not interested in low-carbon tourism.	0.50	0.04	6.65	0.50		
9. I lack travel partners.	0.58	0.03	16.22	0.57		
10. I must consider the physical condition of my partners.	0.56	0.04	5.88	0.50		
11. My family/friends are not interested in low-carbon tourism.	0.51	0.04	6.78	0.76		
12. My family/friends are not interested in low-carbon tourism.	0.51	0.04	8.75	0.62		
13. I have many family obligations.	0.51	0.04	5.07	0.59		
14. The price of low-carbon tourism is unreasonable for me.	0.71	0.03	13.13	0.57		
15. Low-carbon tourism is not convenient enough for me.	0.75	0.03	14.16	0.57		
16. Low-carbon travel is not comfortable enough for me.	0.69	0.03	12.68	0.50		
17. There is insufficient information on low-carbon destinations.	0.59	0.03	10.28	0.58		
18. Less low-carbon tourism products.	0.53	0.04	7.10	0.62		
19. There are fewer operators offering low-carbon tourism.	0.52	0.03	6.99	0.61		
20. Low-carbon tourism content is not attractive.	0.52	0.03	8.80	0.58		
21. I have many other travel alternatives.	0.58	0.03	6.29	0.62		
22. low-carbon tourism has never been a travel option for me.	0.51	0.03	8.76	0.62		
23. low-carbon life is not what I want.	0.54	0.03	9.36	0.66		
**Green commitment**					**0.58**	**0.75**
1. I am a person who endorses a green lifestyle.	0.55	0.05	7.35	0.51		
2. I feel personally invested in protecting the environment.	0.58	0.05	7.94	0.60		
3. I feel guilty if what I do negatively affects the environment.	0.58	0.05	8.05	0.63		
4. I think people should make extra efforts to protect the environment.	0.67	0.05	6.03	0.60		
5. I feel I have enough knowledge to make well-informed decisions on environmental issues.	0.66	0.03	5.85	0.62		
**How frequently have you performed each of the following activities in the past two weeks?**						
6. Gone out of your way to seek out green products (i.e., organic, fair-trade, locally sourced, and sustainable products).	0.66	0.04	13.51	0.57		
7. Recycled and kept your garbage in separate piles of glass, plastic, paper, and metal.	0.52	0.05	8.80	0.62		
8. Read documents online and avoided printouts as much as possible.	0.69	0.03	6.33	0.62		
9. Switched to high efficiency light bulbs.	0.53	0.04	7.03	0.57		
10. Switched to energy efficient appliances.	0.68	0.03	8.03	0.56		
11. Consciously avoided plastic bags and carried reusable shopping bags.	0.51	0.03	6.67	0.58		
12. Took shorter showers and turned the tap off when brushing teeth.	0.50	0.03	8.33	0.52		
**How frequently do you intend to perform each of the following activities soon (i.e., in the next two months)?**						
13. Go out of your way to seek out green products (i.e., organic, fair-trade, locally sourced, and sustainable products)	0.51	0.03	6.67	0.56		
14. Made sure to switch off the lights in unused rooms.	0.58	0.05	9.92	0.62		
15. Read documents online and avoided printouts as much as possible.	0.58	0.05	9.96	0.58		
16. Switch to high efficiency light bulbs.	0.54	0.03	9.08	0.60		
17. Switch to energy efficient appliances.	0.61	0.05	10.73	0.52		
18. Consciously avoid plastic bags and carry reusable shopping bags.	0.54	0.03	9.14	0.62		
19. Take shorter showers and turn the tap off when brushing teeth.	0.55	0.03	9.46	0.51		
**Low-carbon travel intention**					**0.65**	**0.64**
1. I will engage in low-carbon tourism activities in the future.	0.73	0.05	12.54	0.59		
2. I would recommend relatives and friends to engage in low-carbon tourism.	0.69	0.05	11.69	0.53		
3. I consciously have the responsibility to protect the tourism environment.	0.63	0.05	10.45	0.74		
4. I am happy to gain knowledge of low-carbon tourism.	0.58	0.05	9.59	0.72		

**Table 2:** Correlation coefficient matrix (*n* = 358).

	Mean Value	Standard Error	Green Commitment	Low-Carbon Travel Motivation	Low-Carbon Travel Constraint	Low-Carbon Travel Intention
Green commitment	3.94	0.03	**0.76**			
Low-carbon travel motivation	3.91	0.03	0.62 **	**0.79**		
Low-carbon travel constraint	3.18	0.04	−0.17 **	−0.17 **	**0.77**	
Low-carbon travel intention	4.08	0.04	0.63 **	0.65 **	−0.20 **	**0.80**

Note: The value of the positive diagonal line (the diagonal and bold part) represents the root mean square of AVE; the value below it is the standardized correlation coefficient; ** *p* < 0.01.

**Table 3:** Table of estimated parameters of hypothetical paths for theoretical structural models (*n* = 358).

Path	Hypothesis	Standard Coefficient	Standard Error	t-Value
Green commitment to low-carbon travel motivation	H1	0.62 **	0.12	5.21
Green commitment to low-carbon travel constraint	H2	−0.17 *	0.07	−2.40
Green commitment to low-carbon travel intention	H3	0.37 **	0.09	4.26
Low-carbon travel motivation to low-carbon travel intention	H4	0.41 **	0.11	3.82
Low-carbon travel constraint to low-carbon travel intention	H5	−0.07	0.06	−1.15

Note: * *p* < 0.05, ** *p* < 0.01.

**Table 4:** The comparison of the strengths of this study with previous studies.

Source	The Previous Studies
Kousar, et al. [65]	1. Focused student’s environment awareness for air quality and an individual’s understanding of the environmental protection and their actions to save or harm the natural environment.
Kaida and Kaida [66]; Panagiotopoulou, et al. [67]	2. Mentioned climate-friendly behavior and emphasized behaviors that minimize the adverse pressure on ecological conditions with diversified degrees of regularity; 3. Indicated pro-environmental behaviors were an individual’s take “protective actions” towards the environment; 4. Considered the importance of climate and climate change (CCA) to discuss environmental protection behaviors.
Ahmad, et al. [68]; Wang, et al. [69]; Teng and He [70]	5. Emphasized promoting environmental awareness among individuals to improve environmental quality.
Li, et al. [71]; Moody-Marshall [72]	6. Mentioned individual human activities were regarded as responsible for environmental deterioration.
Zeng, et al. [73]; Thor and Karlsudd [74]; Yang, et al. [75]	7. Focused environmental education was used to improve climate change awareness but brings no respect for the environment until our attitudes toward environmental quality improve.
**Source**	**Strengths of the present study**
**Present study**	1. Proposed a diamond model based on the perspective of consumers’ green commitments on low-carbon travel motivation and constraint to improve the low-carbon travel intention; 2. Provided an evidence-based practice study using consumers’ green commitments perspective on low-carbon travel theories for representative cases Weihai and Quanzhou famous tourist cities in China; 3. Developed a questionnaire based on consumers’ green commitments for measuring low-carbon travel attitude, and adding the particularity from **“coastal livable city/world heritage site”** green travel; 4. Remedied research gaps of previous studies, the consumers’ green commitments, and low-carbon travel theories are utilized in this study to explore the relationship between consumers’ green commitments on low-carbon travel motivation and constraint, and the possibility of subsequent low-carbon travel intention; 5. We aimed at low-carbon tourists, who experience green tourist attractions to understand low-carbon travel attitudes.

## Data Availability

Not applicable.

## Appendix A

**Table A1:** Basic personal data.

Items
1. Gender: Male; Female.
2. Age: Less than 21 years old; 21 to 30 years old; 30 to 40 years old; 41 to 50 years old; 51 to 60 years old; more than 60 years old.
3. Education level: Junior high school and below; high school/vocational/technical school; college/university; master’s degree or above.
4. Marital status: unmarried; married but no child; married with children; others.
5. Occupation: Students, family management, self-employed, retired; education, information, government industry; mining industry and manufacturing; agriculture, forestry, fishery and animal husbandry; traditional industry and social service industry.
6. Average monthly income (RMB): Less than 4501; 4501 to 9000; 9001 to 13500; more than 13500.
7. Reduce resource waste.
8. Travel frequency in the past year: 0; 1 time; 2 to 3 times; 4–6 times; more than 6 times.

**Table A2:** The low-carbon travel attitude questionnaire.

Constructs and Items
**Low-carbon travel motivation (low-carbon travel in the “coastal livable city Weihai/world heritage site Quanzhou” would…** **)**
1. Help my health.
2. Reduce the impact on tourism environment.
3. Help me identify the concept of energy-saving and carbon-reducing (ESCR).
4. Help me obtain low-carbon tourism knowledge.
5. Reduce resource waste.
6. Make me interested in the topic of energy saving and carbon reduction.
7. Help me meet people with the same habit in ESCR.
8. Help me respect nature.
9. Allow me to be responsible for the environment.
10. Provide opportunities for family/parental environmental education.
11. Make me stay away from daily life/work environment.
12. Make me avoid daily life/work stress.
13. Make me obtain peace of mind and body.
14. Make me contact my relatives and friends.
15. Make me explore the humanities of travel destinations at a fixed place deeply.
16. Make me experience convenient mass transportation (train, bus, and high-speed rail).
17. Make me experience perfect bicycle or trail facilities.
18. Help the industries have environmental labels (such as environmentally friendly hotels, environmentally friendly restaurants, and environmentally friendly vehicles).
19. Help me to think environmental slogans and appeals of travel industries.
20. Improve travel industries to provide ESCR measures.
21. Help me buy environmentally friendly products.
22. Help me purchase local products.
23. Help me eat local foods.
24. Help me experience the difference between low-carbon tourism and general tourism.
**Low-carbon travel constraint (Not to low-carbon travel in the “coastal livable city Weihai/world heritage site Quanzhou” because…** **)**
1. My low-carbon tourism knowledge is not enough.
2. I do not know how to engage in low-carbon tourism.
3. My physical condition is not suitable for low-carbon tourism.
4. I have no time for low-carbon tourism.
5. I have no money for low-carbon tourism.
6. I have no energy for low-carbon tourism.
7. I have a lot of work or scholarship responsibility.
8. I am not interested in low-carbon tourism.
9. I lack travel partners.
10. I must consider the physical condition of my partners.
11. My family/friends are not interested in low-carbon tourism.
12. My family/friends are not interested in low-carbon tourism.
13. I have many family obligations.
14. The price of low-carbon tourism is unreasonable for me.
15. Low-carbon tourism is not convenient enough for me.
16. Low-carbon travel is not comfortable enough for me.
17. There is insufficient information on low-carbon destinations.
18. Less low-carbon tourism products.
19. There are fewer operators offering low-carbon tourism.
20. Low-carbon tourism content is not attractive.
21. I have many other travel alternatives.
22. Low-carbon tourism has never been a travel option for me.
23. Low-carbon life is not what I want.
**Green commitment**
1. I am a person who endorses a green lifestyle.
2. I feel personally invested in protecting the environment.
3. I feel guilty if what I do negatively affects the environment.
4. I think people should make extra efforts to protect the environment.
5. I feel I have enough knowledge to make well-informed decisions on environmental issues.
**How frequently have you performed each of the following activities in the past two weeks?**
6. Gone out of your way to seek out green products (i.e., organic, fair- trade, locally sourced, and sustainable products).
7. Recycled and kept your garbage in separate piles of glass, plastic, paper, and metal.
8. Read documents online and avoided printouts as much as possible.
9. Switched to high efficiency light bulbs.
10. Switched to energy efficient appliances.
11. Consciously avoided plastic bags and carried reusable shopping bags.
12. Took shorter showers and turned the tap off when brushing teeth.
**How frequently do you intend to perform each of the following activities soon (i.e., in the next two months)?**
13. Go out of your way to seek out green products (i.e., organic, fair- trade, locally sourced, and sustainable products)
14. Made sure to switch off the lights in unused rooms.
15. Read documents online and avoided printouts as much as possible.
16. Switch to high efficiency light bulbs.
17. Switch to energy efficient appliances.
18. Consciously avoid plastic bags and carry reusable shopping bags.
19. Take shorter showers and turn the tap off when brushing teeth.
**Low-carbon travel intention**
1. I will engage in low-carbon tourism activities in the future.
2. I would recommend relatives and friends to engage in low-carbon tourism.
3. I consciously have the responsibility to protect the tourism environment.
4. I am happy to gain knowledge of low-carbon tourism.
